# Trends in vegetation productivity related to climate change in China’s Pearl River Delta

**DOI:** 10.1371/journal.pone.0245467

**Published:** 2021-02-24

**Authors:** Sawaid Abbas, Janet E. Nichol, Man Sing Wong

**Affiliations:** 1 Department of Land Surveying and Geo-informatics, The Hong Kong Polytechnic University, Hong Kong SAR, China; 2 Department of Geography, School of Global Studies, University of Sussex, Falmer Brighton, United Kingdom; 3 Research Institute for Sustainable Urban Development, The Hong Kong Polytechnic University, Hong Kong SAR, China; Italian National Research Council (CNR), ITALY

## Abstract

Climate change will be a powerful stressor on ecosystems and biodiversity in the second half of the 21^st^ century. In this study, we used the satellite-derived Normalized Difference Vegetation Index (NDVI) to examine a 34-year trend along with the response of vegetation to climate indicators surrounding the world’s largest megacity: the Pearl River Delta (PRD) of China. An overall increasing trend is observed in vegetation productivity metrics over the study period 1982 to 2015. Increase in winter productivity in both natural ecosystems and croplands is more related to increasing temperatures (r = 0.5–0.78), than to changes in rainfall. For growing season productivity, negative correlations with temperature were observed in cropland regions, and some forests in the northern part of PRD region, suggesting high-temperature stress on crop production and forest vegetation. However, increased winter and spring temperatures provide higher opportunities for cropping in winter. During the decade 1995–2004, vegetation productivity metrics showed a reversal in the upward trend. The geographical and biological complexity of the region under significant climatic and development impacts suggests causative factors would be synergistic. These include our observed decrease in sunshine hours, increasing cloud cover associated with atmospheric aerosols from industrial and urban development, direct pollution effects on plant growth, and exceedance of high temperature growth thresholds.

## Introduction

The Intergovernmental Panel on Climate Change (IPCC)’s Fifth Assessment Report (AR5), expresses high confidence that climate change will be a powerful stressor on both natural and cultivated ecosystems in the second half of the 21^st^ century [1], especially under high-warming scenarios. The IPCC also cites impacts on global food production such as a potential 10–22% reduction in the Chinese rice yield [2], and decline in tropical plants which are already nearing their upper thermal limits [3]. In most cities of the developing world, the surrounding hinterlands are intensively cultivated to supply urban markets, therefore more significant impacts of climate change on food production would be expected.

In evaluating the impacts of climate change on ecosystems at a global scale, the remote sensing-based Normalized Difference Vegetation Index (NDVI) has been extensively used along with climate data [4–7]. The NDVI shows a high correlation with vegetation productivity; and the effectiveness of NDVI is based on the fact that chlorophyll in healthy green leaves strongly absorbs red radiation, and the spongy mesophyll layer in healthy leaves strongly reflects near infra-red (NIR) radiation [8]. Changes in vegetation phenology and productivity have become an important topic in global climate change and ecosystem analysis research. Changes in phenology are expressed as changes in leaf density and photosynthetic activity through growing seasons [9,10]. Seasonal characteristics of plants, such as emergence and senescence, depending on the characteristics of the lower atmosphere, including the annual cycle of weather patterns and temperature and precipitation characteristics. For example, Jeong et al., (2011) [11] measured the start, end and length of vegetative growth from NDVI time series, compared to climate data over three decades, in the northern hemisphere. They observed that the growing season had advanced by 3.5 days and growing season length increased by 6.5 days per decade. De Jong et al., (2013) [6] used NDVI to examine the factors responsible for greening or browning trends in vegetation activity globally, and found that half of the observed trends were induced by climatic changes.

Recent studies on climate change impacts on flora and fauna in subtropical and tropical areas of China have observed an increased incidence of damage from extreme climate events such as severe spring frosts following unseasonal warm weather which precipitates early tissue growth [12,13]. Ge et al., (2015) [13] concurred that a higher frequency of frost damage would favour deciduous species, which are more resilient to frost and in the long run would cause the disappearance of evergreen forests above 600 m in areas of central China.

As one of the world’s emerging economies, China has experienced rapid development, industrialization, and urbanization. At present, China is dealing with conflicting issues of mitigating climate change in the international arena, while protecting its resources and environment during a domestic socioeconomic transition. Recently 10 megacities have emerged in China, among which the Pearl River Delta (PRD) region is the largest cluster of megacities. Whereas the average global temperature has increased by approximately ~0.8°C since 1880, two-thirds of this since 1975 [14], in the largest cities, the urban heat island (UHI) effect has added 1.72°C to the global increase [15]. In the PRD region temperature increase due to the UHI effect in the 1983 to 1993 decade of rapid industrialization and urbanization was 0.4°C [16], and air quality deteriorated, with photochemical smog and NOx becoming semi-persistent over the region [17]. In addition, the regional heat dome circulation over PRD suggests local temperatures will increase further, even without further local developments [18]. Accompanying these climatic effects have been dramatic forest and vegetation disturbances, soil erosion, and loss of farmlands, natural ecosystems and biodiversity.

Many site-specific ecological studies have been conducted in the PRD region, but the impacts of changes in meteorological parameters e.g. seasonal metrics of temperature, precipitation as well as landscape-wide climatic sensitivity, have not been studied. Most climate change impact studies address either natural or human-modified ecosystems. This study focusses on determining vegetation responses to different climatic factors across a large, dynamic and diverse region of rural-to-urban transition. The study determines the spatial patterns of temporal trends in precipitation, temperature, and vegetation phenology and productivity.

### Study area

**Fig 1:**
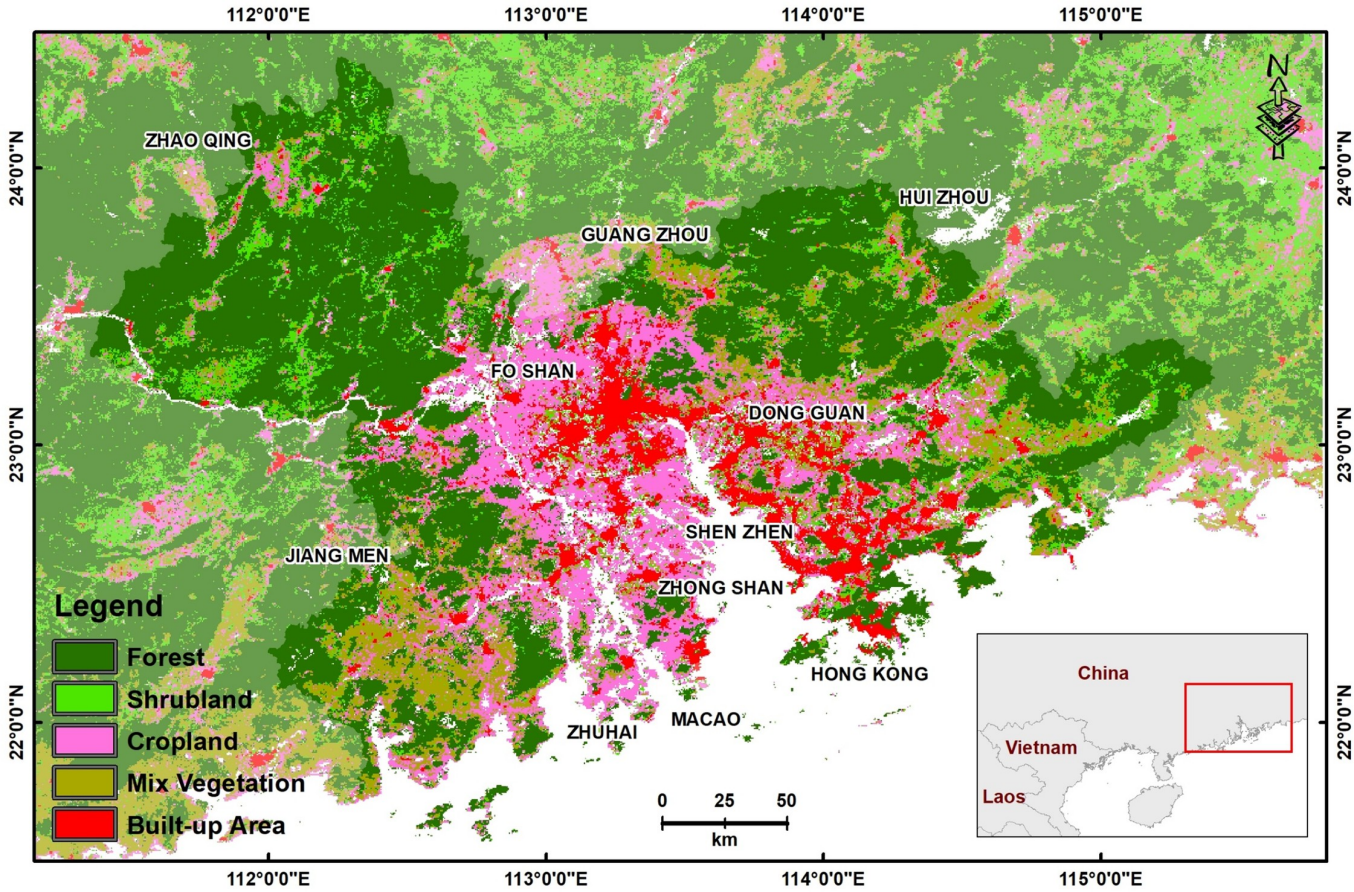
Location map of the study area and spatial distribution of land use land cover classes [21].

The PRD region has grown at a breakneck pace, from farmland to urban-industrial in the last three decades. Although the PRD region contains eight megacities with populations over 10 million and 123 cities with between 1 and 10 million, almost 63% of the population was still non-urban in 2015 [19]. If all PRD cities are considered as one entity, the PRD overtook Tokyo as the world’s largest megacity in both size and population, in 2010 [20]. Accompanying this growth has been a dramatic deterioration in air quality, although no data indicating long-term trends are available. The study area comprises the 11 PRD prefectures of Hong Kong, Macau, Shenzhen, Guangzhou, Dongguan, Shunde, Jiangmen, Foshan, Zhongshan, Huizhou and Zhaoquing (Fig 1). A land use land cover (LULC) map from the Moderate Resolution Imaging Spectroradiometer (MODIS) satellite images indicate approximately 9% urban/built-up, 31% cropland including tree cultivation, and 45% forest (Fig 1). Rice, with two crops a year occupies 76% of agricultural land, with the early crop from March to July and the late crop from July to November. Often this will be followed by a vegetable, winter wheat, or rapeseed. In addition, much of China’s output of sugarcane is produced in the region. Other agricultural products include sweet potato, vegetables, citrus fruits and tea. With increasing industrialization, more land has been converted to cash crops and vegetables, and grain production has intensified recently. This small PRD region, only 0.4% of China’s land area, now accounts for 10% of China’s gross domestic product (GDP). The climate of the PRD is tropical, with hot and humid summers from June to August, with approximately 2500 mm rainfall between April and September, and cool dry winters from November to March. The natural vegetation consists of tropical and subtropical evergreen forest.

## Methods

### Data sets and pre-processing

#### NDVI data and phenology extraction

Coarse-scale GIMMS (Global Inventory Modeling and Mapping Studies) NDVI datasets provide the most consistent long-term data of the earth’s biosphere [22]. Its recent version (NDVI3g.v1; https://ecocast.arc.nasa.gov/data/pub/gimms/3g.v1/) [23,24] spans more than 34 years from 1981–2015 at 8 km spatial resolution (Table 1). The datasets are based on 15-day maximum value composites that provide 24 NDVI composite images a year. For this study, we used 816 fortnightly observations for 34 years (1982 to 2015) of the NDVI data. The fortnightly Maximum Value Composites (MVC) largely removed noise, though it includes disturbances caused by cloud contamination, atmospheric variability, and bidirectional effects, which show as undesirable noise [5], therefore, the NDVI product is accompanied by a quality assessment (QA) layer which enables pixels to be excluded or weighted when reconstructing the time series. To avoid this, the Whittaker smoothing approach was implemented in R to reconstruct a continuous NDVI time-series.

**Table 1:** Datasets used for the study.

No.	Data/Product	Data Type	Time Range	Spatial Resolution	Temporal Resolution
1	NDVI GIMMS3g [23,24]	Raster	1982–2015	~8 km	15 Days
2	CHIRPS Precipitation [25]	Raster	1982–2015	~5 km	Daily
3	ERA5 (mean_2m_air_temperature) [26]	Raster	1982–2015	~25 km	Daily
4	MODIS LCLU product (MCD12Q1) [21]	Raster	2001–2015	~0.5 km	Annual

In evergreen ecosystems, changes in vegetation phenology and productivity are driven by the emergence of new leaves and shedding of older leaves, whereas seasonality in photosynthesis is explained by leaf development and demography [27]. We applied the midpoint pixel approach to extract the Start of Season (SOS) and End of Season (EOS) time from the smoothed NDVI time series by scaling the annual cycle between 0 and 1 [28]. This is one of the most consistent methods for extraction of phenology metrics regardless of vegetation cover type, and applicable to a variety of ecosystems [7,29]. Then six additional phenology and vegetation productivity metrics (length of growing season (LOS), position of season peak (POP), position of season trough (POT), mean growing season NDVI (MGS), maximum seasonal NDVI (Peak), and minimum seasonal NDVI (Trough)) were determined using the PhenologyRaster() function in the R package ‘green-brown’. Further analysis was confined to pixels showing significant seasonality where the mean annual NDVI amplitude was greater than 0.1 [29]. Description and ecological meanings of the phenometrics are given in the supplementary S1 Table.

#### Seasonal climate variables from satellite-based precipitation and temperature data

Temperature and precipitation are considered representative climate variables. Station-based point measurements cannot capture a continuous spatial distribution of precipitation and temperature [30]. Therefore, to compare the spatial and temporal variability of precipitation with the NDVI data, the CHIRPS (Climate Hazards Group Infrared Precipitation with Station) data set, developed by the United States Geological Survey (USGS) in collaboration with Earth Resource Observation and Science (EROS) centre is used [25]. CHIRPS is generated by integrating satellite imagery and *in-situ* gauge collected observations. The daily rainfall data are distributed at 0.5° (~5 km) spatial resolution. In this study, we used the daily CHIRPS data from 1981 to 2015. Similarly, temperature data were derived from the gridded ERA5, the 5^th^ generation Reanalysis data of the European Centre for Medium-Range Weather Forecasts (ECMWF), temperature product (mean_2m_air_temperature) provided by the Copernicus Climate Change Service (C3S) [26]. The daily average temperature datasets are available from 1979 to ‘within 5 days of real time’ at a spatial resolution of 0.25°. To examine the functional relationship between the climate variables and the phenology metrics as well as to explain the year-to-year variation in vegetation dynamics, the climate variables (temperature and precipitation) were converted to seasonal and annual composites. Layers of annual and seasonal (four seasons) variables for precipitation (accumulative) and temperature (average) were created. The winter season comprises December, January, and February (DJF), spring comprises March, April, May (MAM); summer is June, July and August (JJA), and autumn includes September, October and November (SON).

#### Land use land cover data

The LULC map of the study area was prepared from a MODIS land cover type product (MCD12Q1, version 6 product) for 2001 and 2015, at 500 m spatial resolution. The data was retrieved from the NASA EOSDIS Land Processes Distributed Active Archive Center (LP DAAC, https://lpdaac.usgs.gov). Both LULC maps of 2001 and 2015 were overlaid to remove pixels showing change over the period, and the remaining persistent pixels were resampled to 8 km resolution using a majority filter to match the spatial resolution of the GIMMS NDVI data. The analysis was performed on pure pixels i.e. those of the 8 km pixels having over 80% of internal sub-pixels belonging to the same class [31], out of the four generalized vegetation classes in the study area, viz., Forest (F), Shrubland (SH), Cropland (CL), and mosaic of Farmland/Cropland/Forest (Mix).

### Temporal trend in phenology and climatology

Temporal trends in phenology metrics over the 34 years 1982–2015, were determined on a pixel by pixel basis. The trends in NDVI series were determined using linear regression on seasonally adjusted time-series as well as on annually aggregated time series [32]. The values of slope (trend) were masked where the p-value was greater than 0.05 (95% confidence level) to obtain only significant NDVI time series trends. For a comprehensive overview of the LULC specific changes in the vegetation and climate variables, all variables were spatially averaged over the LULC classes and linearly regressed along time. In addition to LULC classes, the variables were also averaged for pixels indicating greening and browning trends over time. From the fitted linear models, correlation coefficients, slope, the significance of slope and the total amount of change (multiplying slope of the fitted model by the length of the time series) were determined [6]. These trends were also fitted using a Local Polynomial Regression Fitting (loess) algorithm to show the overall changes over time.

### Spatial correlation of climate variables with seasonal phenology metrics

The functional relationships between NDVI-based phenology metrics and the ten environmental variables (seasonal and annual measures of precipitation and temperature) were examined to explore inter-annual variation in phenology. Partial correlation analysis was used to explain the variation in each phenology metric against changes in temperature and precipitation variables. Thus the confounding effects of temperature were eliminated while computing the partial correlation with each of the precipitation variables, and vice versa for temperature [33]. Insignificant pixels (p > 0.05) were masked out. The results of partial correlations analysis were also spatially aggregated by homogenous patches of LULC classes to understand the vegetation responses across different LULC classes.

### Lag time analysis

The lag times of NDVI to precipitation and temperature were determined by performing correlation analysis. For this, the datasets were transformed to Vegetation Condition Index (VCI) [34], Precipitation Condition Index (PCI) [35], and Temperature Condition Index (TCI) (Eqs 1–3). These transformations are pixel-based normalization of the datasets to control local differences for an integrated raster-based analysis in which short signal of changes in the variables are filtered by separating them from the long-term ecological and climate signals. The cross-correlation coefficients of VCI with PCI and TCI were determined using Pearson’s cross-correlation function (Eq 4) at different lag-time scales (0, 15, 30, …, 150 days). The analysis was performed up to the 10th lag, in 15-day intervals up to 150 days [36].

For each lag, two rasters comprising correlation coefficients and significance values of the correlation were obtained. Each correlation raster layer was masked by pixels with significant correlation (p < 0.05), and insignificant pixels were removed for subsequent analysis. Overlay analysis of all the correlation raster layers was performed to find the highest correlation value of each pixel, then gave the lag time corresponding to the maximum correlation value for every pixel [37]. This resulted in two layers representing maximum correlation, as well as lag time for the maximum correlation.

$$VCI=\frac{\left(NDVI-NDVI_{min}\right)}{\left(NDVI_{max}-NDVI_{min}\right)}$$ (1)

$$PCI=\frac{\left(Rainfall-Rainfall_{min}\right)}{\left(Rainfall_{max}-Rainfall_{min}\right)}$$ (2)

$$TCI=\frac{\left(Temperature-Temperature_{min}\right)}{\left(Temperature_{max}-Temperature_{min}\right)}$$ (3)

where max and min represent the maximum and minimum values of corresponding variables during the study period.

$$PCC\text{=}\frac{\sum_{t=0}^{L-1}\left[\left(TS_{t}^{a}-{\bar{TS}}_{t}^{a}\right)*\left(TS_{t-lag}^{b}-{\bar{TS}}_{}^{b}\right)\right]}{{\left(\sum_{t=0}^{L-1}{\left[\left(TS_{t}^{a}-\bar{TS_{t}^{a}}\right)\right]}^{2}\right)}^{0.5}*{\left(\sum_{t=0}^{L-1}{\left[\left(TS_{t-lag}^{b}-{\bar{TS}}_{}^{b}\right)\right]}^{2}\right)}^{0.5}}$$ (4)

where ${TS}_{t}^{a}$ and ${TS}_{t}^{b}$ b corresponds to VCI and PCI (or TCI), respectively; L is the length of time series, lag is the length of temporal shift applied before the correlation, and t refers to the single time step of the time series.

## Results

### Spatial differences in trends in annual temperature and rainfall during 1982–2015

**Fig 2:**
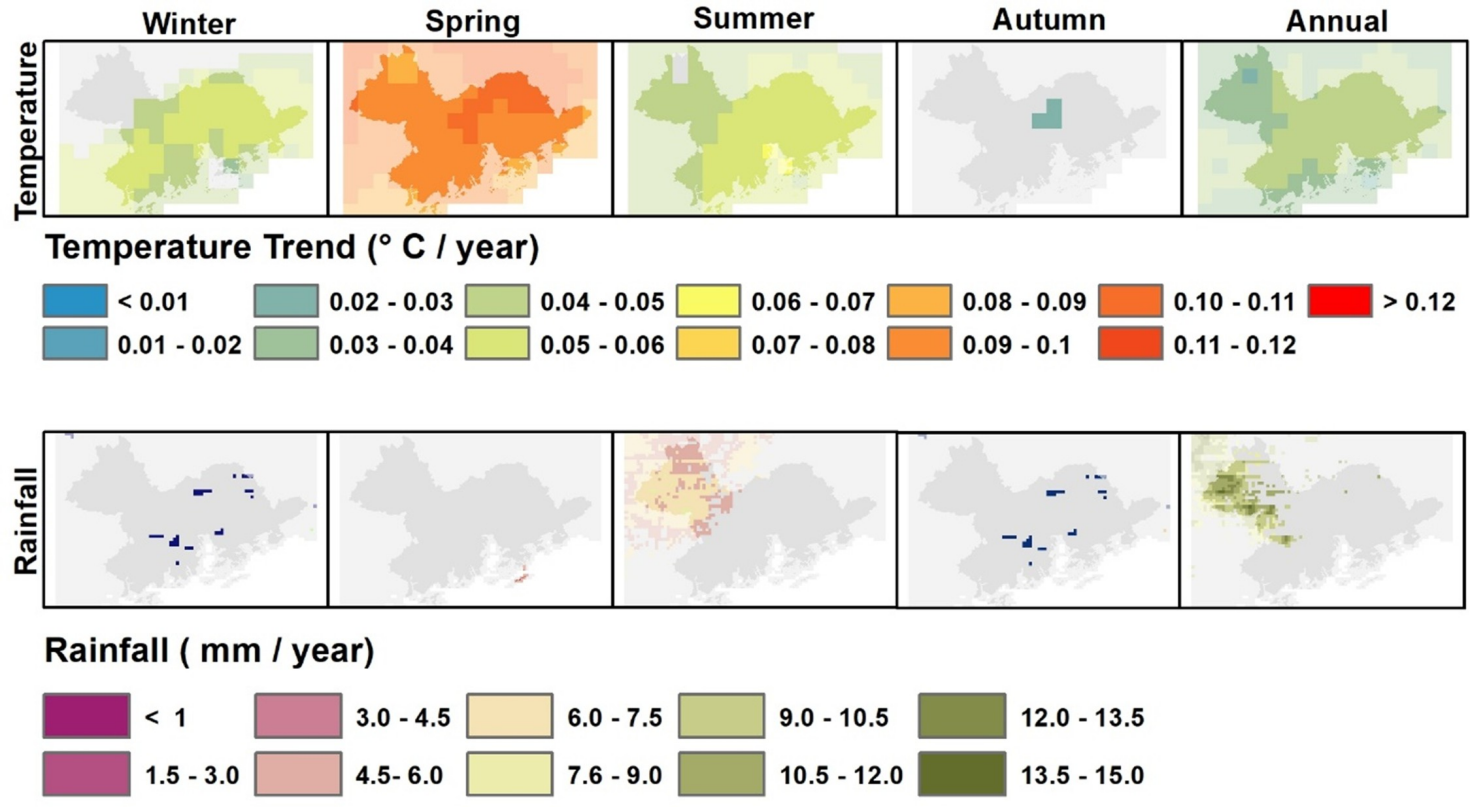
Spatial patterns of trends in seasonal and annual temperature and rainfall. Insignificant pixels (p > 0.05) are masked out in grey.

Temporal trend maps of temperature and rainfall indicate that over the study period, the whole PRD region has undergone a significant increase in temperature, with an increase in mean annual temperature of 0.05–0.06°C per year, amounting to 1–1.2°C over the study period (Fig 2). Spring temperatures show the greatest increase, of almost 2°C. Winter and summer temperatures show an increase of approximately 0.7°C over the study period. Rainfall, on the contrary, shows little change except in the northeast forested region of ZhaoQuing where a small increase is observed over the whole 34 years study period. These image data showing temperature increase distributed evenly over the whole PRD region, are supported by climate station temperature data (S1 Fig) and the rainfall observations are supported by a recent study by Nguyen et al. (2018) [38], who did not observe any significant rainfall trend in this region of China.

### Rapid urban sprawl, deforestation and browning trends

**Fig 3:**
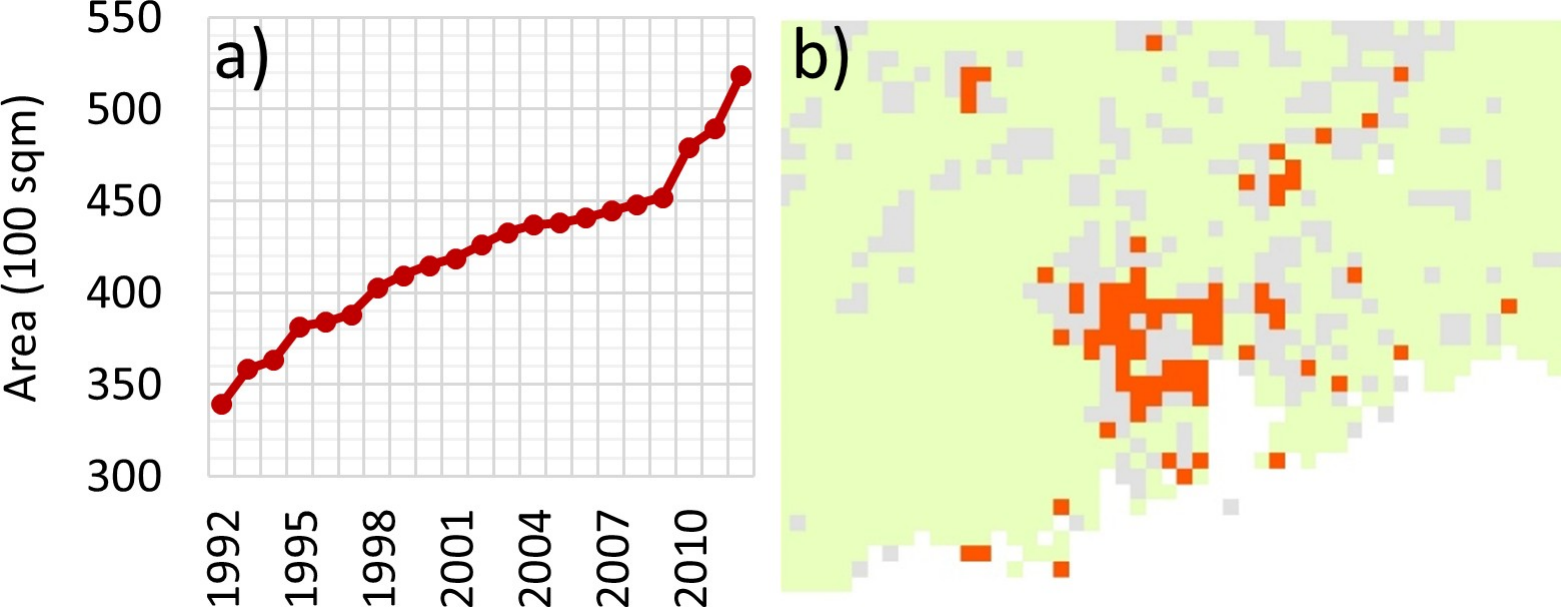
Urbanization and farmland reclamation. a) trend of urban area increase (1992–2012) in the PRD based on nightlight imagery b) annual trend of seasonally aggregated NDVI time series. Orange represents browning pixels while greening pixels are in green, and grey colour shows pixels with insignificant trends (p > 0.05).

Synchronous to these climatic changes has been a dramatic increase in urbanization in the PRD region. Nighttime lights imagery from the NASA’s National Polar-orbiting Partnership satellite show an increase of 340 to 519 million km^2^ in illuminated areas in just 20 years from 1992 to 2012 (Fig 3A). This rapid urbanization trend is also evident from browning trends in NDVI and vegetation productivity metrics (Fig 3B and S2 and S3 Figs). Over the past three decades, urban areas in the PRD region quadrupled, with the accelerated growth of small towns into cities and megacities [39]. The amount of urban area grew from 0.5% in 1979 to 10.8% in 2009 [40] by conversion of cropland into urban areas. Displacement of these farmlands to relatively infertile hilly land away from city centers also resulted in accelerated deforestation [39,40]. On the maps, these deforestation patches are indicated by pixels showing browning trends (Fig 3B). These regions are adjacent to the urban areas of Huizhou, Dongguan and Guangzhou, which themselves show significant urban heat island effects [41] in addition to greenhouse-induced warming. This may partly explain the up to 2°C increase in temperatures noted above, which is well above the global background warming rate of approximately 0.7°C.

### Trends in land cover specific vegetation productivity

**Fig 4:**
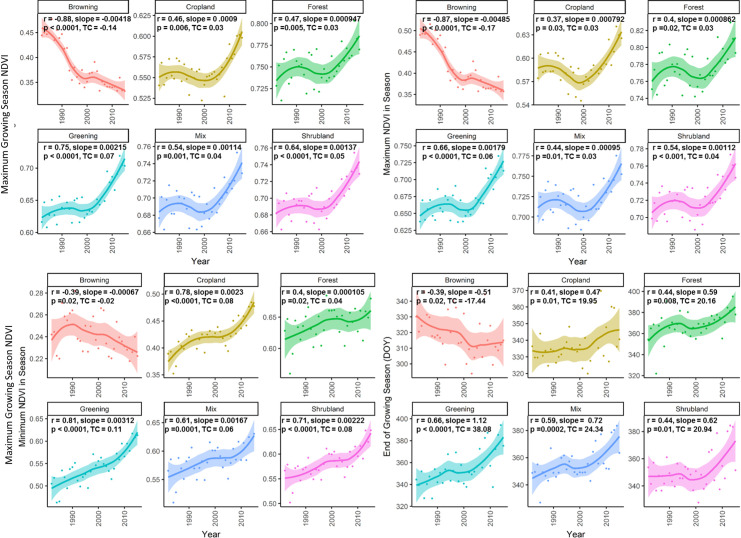
Trends in phenometrics of different LCLU classes with loess fitting and confidence intervals. Only the metrics showing significant trends in most land cover classes; correlation coefficient (r), slope, significance (p) and total change (TC) is obtained by linear regression analysis of the variables over time.

Fig 4 shows the trends of the phenology and productivity metrics, grouped by the individual LULC class, for those metrics showing significant trends over the study period (Table 2). These are the Mean Growing Season NDVI (MGS), the maximum NDVI in the season (Peak), and the minimum NDVI in the season (Trough). When measured over the whole 1982–2015 period, all have increased, and the End of Season (EOS) has become significantly delayed. Spatial distribution of trends in monthly NDVI and phenometrics, as well as graphical summaries of phenometrics, are given in S2–S4 Figs.

**Table 2:** Summary statistics of trends in phenometrics according to LULC class, greening, and browning pixels.

Metric	Class	r	slope	p-value	TC	Metric	Class	r	slope	p-value	TC
**Mean Growing Season (MGS)**	Greening *	0.75	0.0022	0.0000	0.07	**Position of Trough (POT)**	Greening*	0.31	0.370	0.0726	12.58
	Browning*	-0.88	-0.0042	0.0886	-0.14		Browning*	0.34	0.560	0.0465	19.04
	Forest*	0.47	0.0009	0.0053	0.03		Forest	-0.03	-0.040	0.8620	-1.37
	Mixed*	0.54	0.0011	0.0010	0.04		Mixed	0.21	0.244	0.2400	8.30
	Cropland*	0.46	0.0009	0.0064	0.03		Cropland*	0.54	0.617	0.0009	20.98
	Shrubland*	0.64	0.0014	0.0000	0.05		Shrubland	0.23	0.280	0.1870	9.52
**Maximum NDVI in Season (Peak)**	Greening *	0.66	0.0018	0.0000	0.06	**Start of Season**	Greening	0.16	0.192	0.3690	6.53
	Browning*	-0.87	-0.0049	0.0674	-0.16		Browning*	0.30	0.363	0.0799	12.34
	Forest*	0.4	0.0009	0.0206	0.03		Forest	-0.16	-0.225	0.3740	-7.65
	Mixed*	0.44	0.0009	0.0100	0.03		Mixed	-0.04	-0.037	0.8460	-1.27
	Cropland*	0.37	0.0008	0.0316	0.03		Cropland*	0.37	0.303	0.0329	10.30
	Shrubland*	0.54	0.0011	0.0010	0.04		Shrubland	0.06	0.072	0.7410	2.43
**Maximum NDVI in Season (Trough)**	Greening*	0.81	0.0031	0.0000	0.11	**End of Season (EOS)**	Greening*	0.66	1.120	0.0000	38.08
	Browning*	-0.39	-0.0007	0.0233	-0.02		Browning*	-0.39	-0.513	0.0237	-17.44
	Forest*	0.4	0.0011	0.0207	0.04		Forest*	0.44	0.593	0.0085	20.16
	Mixed*	0.61	0.0017	0.0001	0.06		Mixed*	0.59	0.716	0.0002	24.34
	Cropland*	0.78	0.0023	0.0000	0.08		Cropland*	0.41	0.469	0.0169	15.95
	Shrubland*	0.71	0.0022	0.0000	0.08		Shrubland*	0.44	0.616	0.0101	20.94
**Position of Peak (POP)**	Greening	0.01	0.0055	0.9820	0.19	**Length of Season (LOS)**	Greening	0.17	0.238	0.3460	8.09
	Browning*	-0.43	-0.5790	0.0119	-19.69		Browning*	-0.50	-1.010	0.0024	-34.34
	Forest	-0.09	-0.1460	0.6070	-4.96		Forest	0.11	0.141	0.5390	4.79
	Mixed	-0.05	-0.0589	0.7930	-2.00		Mixed	0.14	0.144	0.4330	4.90
	Cropland	-0.04	-0.0349	0.8140	-1.19		Cropland	-0.17	-0.191	0.3390	-6.49
	Shrubland	-0.06	-0.0788	0.7460	-2.68		Shrubland	-0.03	-0.0474	0.8680	-1.61

TC shows total change obtained by multiplying slope of the fitted model by the length of the time series).

* denotes a significant trend.

For cropland, MGS is the most meaningful indicator of crop production and greenness, and this has increased with a significant upward trend of NDVI from 0.55 to 0.62, (r = 0.46) during 1982–2015 (Fig 4). Notably, Trough for cropland areas has increased very significantly from 0.38 to 0.48 (r = 0.78) (Fig 4), suggesting increasing opportunities for crops to be grown in some areas in the winter season.

Both forest and cropland show significantly increasing NDVI for those phenology variables MGS, Peak and Trough, which measure vegetation greenness and/or productivity. Both also show significant lengthening of the growing season over the study period, with a delayed EOS by 15–20 days (all ecosystem types), with advanced SOS by 6–7 days (for forest) and advanced SOS by 10 days for cropland (Table 2). The effective EOS in the southern China region is February to March, the coldest month and dry season. As discussed above (Fig 2), winter and spring temperatures have increased significantly thus these increased winter and spring temperatures are already significantly affecting vegetation productivity of the PRD region.

**Fig 5:**
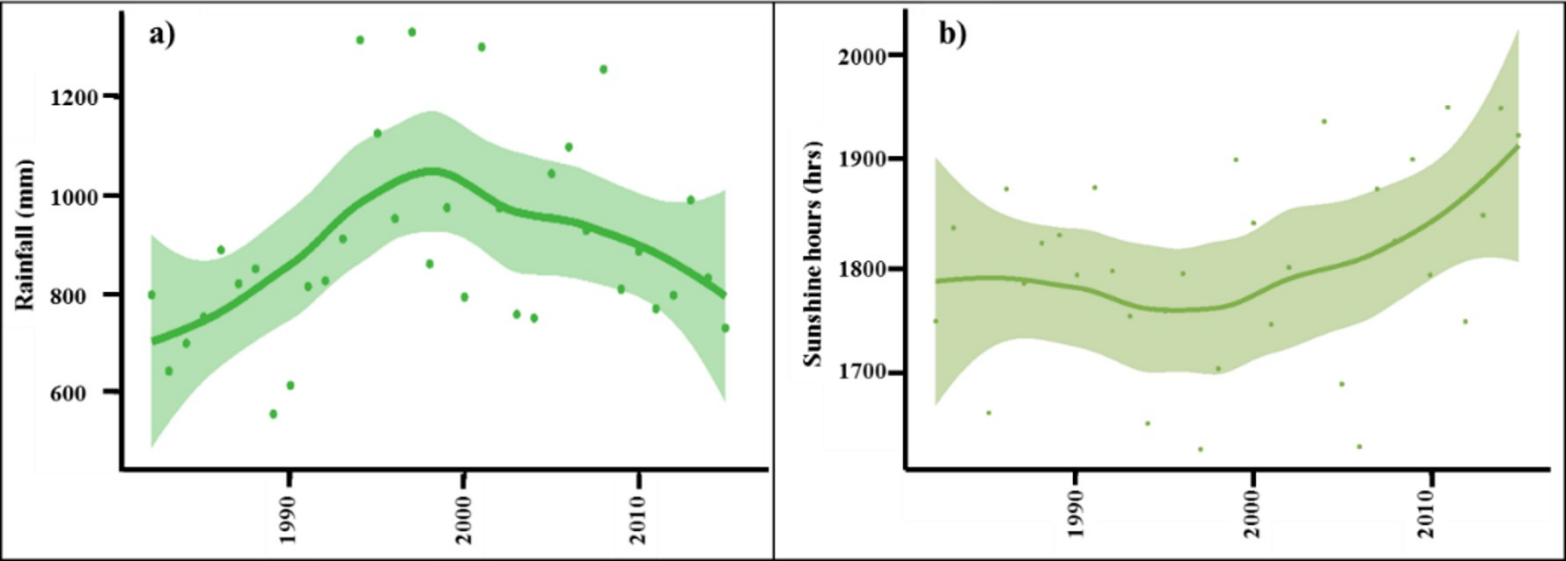
Trends of summer rainfall and sunshine hours in the PRD. (a) Spatially averaged summer rainfall, (b) annual average time series of sunshine hours.

The loess fitting of these four significant phenology metrics (Fig 4) shows an interesting reversal in the upward trend for the NDVI variables MGS and Peak, for all ecosystem types in the middle period from 1995 to 2004, followed by a continued rise up to 2015. It is also notable that the the Maximum NDVI in season Peak shows a much greater trend reversal than the Minimum NDVI in season Trough, suggesting that the reduced NDVI in this middle period 1995 to 2004, may be related to summer, rather than winter conditions. The time of Peak NDVI (POP) for all ecosystem types was also delayed by approximately 5 days in the middle period (Table 1, S5 Fig). Fig 5B shows the loess fitting of the average trend of sunshine hours in the PRD region, which also indicates a significant decline during the late 1990s and early 2000s. This was accompanied by increased summer rain (Fig 5A), and by a period of increased cloudiness over China [42] and southwest China [43]. A decrease in sunshine hours reported for China between 1960–1990 [44] was found to be greater over urban areas. The loess graphs also show increased winter and spring temperatures in the 1995–2004 period, and this is usually consistent with increased cloud cover due to the insulating effects of clouds in cold weather.

### Response of vegetation to seasonal and annual changes in precipitation and temperature

**Fig 6:**
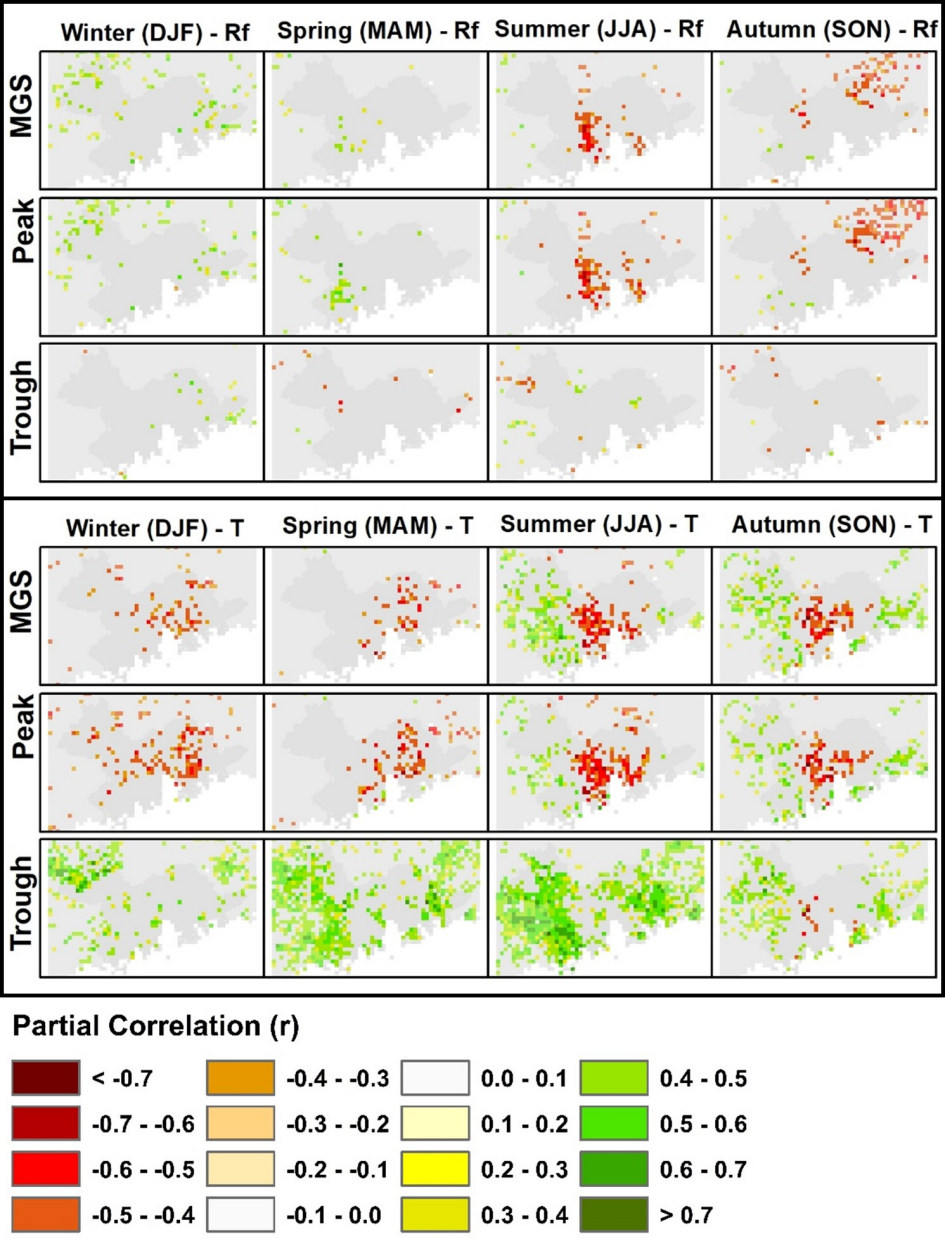
Maps of partial correlation of temperature and rainfall with vegetation productivity metrics by season. Pixels with significant correlation (p ≤ 0.05) are shown while insignificant pixels are removed.

As the NDVI is expected to be related to both temperature and rainfall, we performed partial correlation analysis to control the confounding effects. As most of the mapped variables were not significant (S6 and S7 Figs), only significant variables are shown in Fig 6. It is interesting that the MGS shows a moderate (r = ~-0.6) negative correlation with temperature, especially, but not confined to, crop-growing areas, for all seasons of the year. Peak NDVI also shows a negative correlation with temperature (r = ~-0.6) over cropland as well as over some forested regions for all seasons. On the other hand, Trough (NDVI in February) in both forest and shrubland regions is positively correlated with temperature (r = 0.5–0.7) for all seasons of the year. This significant positive correlation of ecosystem productivity with temperature in the non-growing season (Trough) represents a positive impact on forest growth from the increased winter temperatures in the PRD region as noted above. For growing season, on the other hand, the negative correlations of cropland with temperature for growing season MGS and Peak NDVI are difficult to explain, especially given overall greening trends over the study period.

### Lag time analysis of NDVI with precipitation and temperature

**Fig 7:**
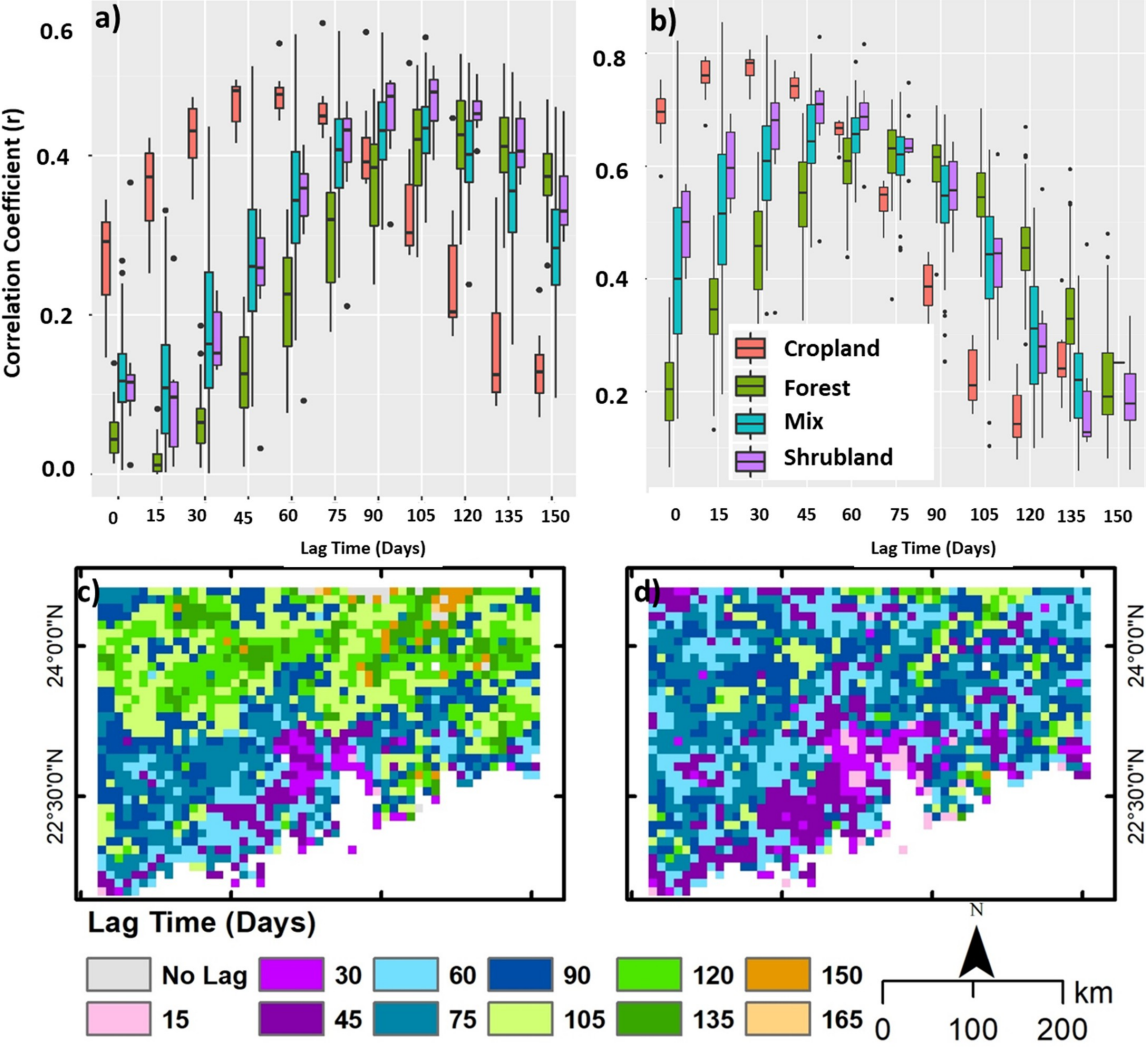
Lag time of NDVI to precipitation and temperature. Lag times for (a) rainfall and (b) temperature to NDVI, of different LUCL classes, the pixel-wise lag time for maximum correlation of (c) rainfall and (d) temperature with NDVI.

In correlating the NDVI time series with temperature and rainfall, correlations were calculated for different lag times for each ecosystem type, in 15-day intervals up to 150 days. Results represent the sensitivity of vegetation productivity to changes in the respective climatic variable on a seasonal basis, and this would suggest the potential time frame of vegetative response to an extreme event such as drought, heatwave or extreme cold. In all ecosystem types, significant lag times are observed (Fig 7), although those for cropland will be strongly influenced by planting patterns and the initially lower vegetation productivity. Cropland shows higher responses to both rainfall and temperature, than do the natural ecosystems, no doubt because planting is timed to coincide with a favourable climate, and the lag times for cropland are no doubt controlled by the time taken for crop development under the favourable rainfall and temperature regimes. Cropland shows a higher response to temperature (r = 0.77) and shorter lag time (30 days), than rainfall (r = 0.48) and 60 days lag time. The natural ecosystems also show a higher response to temperature, with a 75 days lag time for forest (r = 0.63) than to rainfall with 120 days for forest (r = 0.43). Shrubland’s response is approximately between those of forest and cropland in both the level of correlation as well as lag time. The natural ecosystems show almost no response to present rainfall ie. zero lag time, (r < 0.15), but their response to temperature at zero lag time is higher, ranging r between 0.2 and 0.4.

## Discussion

This study demonstrates significant medium-term changes in vegetation productivity over the region incorporating the world’s largest megacity, the PRD region of southern China. We observed increasing productivity in both natural and agricultural ecosystems over the 34-year period 34 1982–2015, especially in the winter season. However, a reversal in the upward trend for seasonal climate indicators MGS and Peak for the middle period between 1995 and 2004 suggests the operation of factors other than temperature.

The observed negative correlations between cropland and summer-time temperatures accord with warnings [2,45] that rising global temperatures may impose stress on tropical crops grown in summer such as rice, which occupies 76% of agricultural land in PRD. The overall greening trend may also be associated with other factors such as CO_2_ fertilisation or intensified farming. However, the observed increased in warming (Fig 2) and its negative influence on cropland greening and positive association with natural vegetation (Figs 4 and 6) is profound and corresponds with other global and regional studies [2,45–47]. For vegetation growth and high grain yield, rice needs daytime air temperatures between 25°C and 32°C, but lower temperatures between 20°C and 25°C for grain filling and ripening [48]. However, summer daytime temperatures in the region have recently exceeded this regularly, as mean daily temperatures for June to September in Hong Kong are now around 28°C [49]. Rice is Asia’s foremost staple food crop, and climate change predictions for rice production vary according to models used as well as with or without CO_2_ fertilization effects [50]. Wassmann et al. (2009) [51] explained that in many parts of Asia including China, current temperatures in July and August are already approaching critical levels for rice production during the critical growth stages, and this is supported by Tao et al. (2013) [52], who found that rice yield in eastern China was reduced by 3.4% due to increase in mean temperature during the vegetation growth period. Furthermore, Tao and Zhang (2013) [47] explained that rising temperatures accelerate the growth of rice, leaving less time for grain development. Lesk et al. (2016) [53] found that extreme temperature stress during the growing season had reduced global cereal yields by 9–10% between 1964 and 2007, thus our finding should not be unexpected. It is important to note that the crop greening or extension of growing season may not necessarily show a direct correlation with crop yield, especially under mixed or heterogenous cropping system [54]. Crop yield may vary non-linearly with increasing NDVI or greenness indicators as NDVI may not indicate a direct relation with crop biophysical parameters [55]. For example, the relationship between NDVI and Leaf Area Index (LAI) becomes non-linear in crops with highly dense canopy [56].

Besides croplands, some forest and shrubland regions in the northern parts of Huizhou and Guangzhou prefectures also show a negative correlation with temperature (r = -0.6), and the dissociation between temperature and forest greening has been noted in other studies. For example, deciduous needle-leaf forest in Russia showed negative response to warming [46] which is counter-intuitive to the observed increase in plant growth in northern high latitudes due to rising temperatures [46,57–59]. Guo et al. (2018) [57] showed that between 1982 and 2012, only 20% of the non-tropical northern hemisphere showed a strong positive relationship between temperature and NDVI, which shrunk from 32% in 1982, and NDVI trends were supported by tree ring evidence. Since nowhere in the PRD region is too cold for plant growth, rising temperatures would not necessarily generate increased productivity, and the observed negative correlations may, as suggested by Corlett, (2011) [3] be a sign of an upper temperature threshold being reached. These may be reached earlier under conditions of moisture stress from increased evapotranspiration in a warming climate. The result is reduced carbon uptake with decreased productivity, rather than direct damage to leaf tissues.

The correlation and lag time maps could provide inputs in developing specific models for vegetation response to climate predictors by considering temporal lag dynamics. For example, during the spring drought during 2009–10 in Yunnan, the southwestern province of China, it was observed that lag time response of vegetation to rainfall deficiency was 90 days for the evergreen forest while Cropland and Shrubland became stressed earlier (64 days). This indicates varying resistance capacities of different vegetation regimes in an ecosystem [60,61]. However, resistance to extreme climate events may also change due to species composition of plant communities within a particular land cover type. For example, Abbas et al., (2017) [12] observed that tropical species suffered more than sub-tropical species during the extreme cold event in January 2016 in the degraded tropical secondary forest of Hong Kong. Apart from temporal lag, vegetation response to climate variability can also vary in an ecosystem. For example, Bégué et al. (2011) [62] divided the study area into three eco-climatic zones based on the NDVI and rainfall relationship (positive, negative and neutral). Thus, the nature of vegetation response to climate variability and inputs from lag time maps may enhance mapping and understanding of intricate responses of dynamic land cover regimes to climate indicators.

The observed reversal in the upward trend in productivity during the decade 1995–2004 corresponds to a period of rapid urbanization and industrialization, deteriorating air quality and exceptionally warm years. Studies from other regions using GIMMS NDVI have also reported a reversal in greening trends around the same period, from the mid-1990s to mid-2000s. For example, Liu et al (2005) [63] found trends of greening-browning-greening for the periods 1982–1994, 1995–2004, and 2005–2012 respectively over the globe, with browning in the middle period in many regions. While they do not provide any overall explanation, other researchers working in specific regions give climatic or other explanations. For example, Park and Sohn (2010) [64] observed a browning period from 1997 to 2006 in northern East Asia around 50°N, explained as reduced rainfall combined with continued warming creating moisture stress. Piao et al. (2014) [65] also attributed a weakened relationship between temperature and productivity after 1997 in northern Eurasia, to increased drought under rising temperatures. Weakening relationships between productivity and temperature over recent decades were also observed by de Jong et al. (2013) [46], attributed to large scale climatic catastrophes accompanying climate change. The complexity of the PRD region precludes a simple explanation for a decadal decline in productivity. However, ongoing massive urbanization and industrialization in the PRD during the study period suggests that our observations of declining sunshine hours in the late 1990s to early 2000s are at least partly due to dimming from atmospheric aerosols. Higher temperatures during this period may also be partly responsible for the productivity decline, as agricultural and forest plants meet upper temperature thresholds. This is supported by our correlation analysis (section 3.4), with negative correlations between temperature and MGS and Peak. In particular, the main staple crop rice may not support the higher summer temperatures observed over recent decades.

Others have noted trends in solar radiation, rather than in temperature and rainfall, affecting plant productivity. Nemani et al., (2003) [66] observed an increase in Net Primary Productivity (NPP) in tropical ecosystems between 1982 and 1999, which was primarily attributed to decreased cloudiness and increased solar radiation. Wei et al. (2018) [67] found that reduced radiation, rather than temperature or rainfall anomalies, explains reversals in greenness in North Central Asia. Piao et al. (2013) [68] found that solar radiation limited productivity in the northern regions. In addition to increased cloud cover, our observed reduced sunshine hours from the late 1990s to early 2000s may be associated with the PRD region’s accelerated urbanization and industrial development especially during the 1995 to 2004 period [69] with low efficiency in energy use, and consequently increased aerosols/pollution [43,44]. Wang et al. (2017) [44] found that in China, the ratio of rural to urban dimming increased from 0.39 to 0.87 with increasing urbanization, and reached a maximum when urbanization reached 50% or population exceeded 250 persons/km^2^. In PRD region, air pollution produces a fog-like haze, which reflects and absorbs solar radiation, and this effect has been reported from other parts of China [70,71] and Asia [72,73].

Therefore, the reduction in vegetation productivity observed during the late 1990s and early 2000s observed here could, in addition to reduced sunshine hours due to increased cloud cover, result from dimming due to aerosols (Fig 5A). Aerosols act as condensation nuclei in cloud formation and may increase rainfall (Fig 5B). Aerosols also scatter sunlight, enhancing planetary albedo, thus reducing received radiation. Additionally, exposure to high surface ozone and SO_2_ concentrations common in the PRD [74] can stunt or damage plants [75]. Indeed, tree ring analysis of *Pinus massoniana*, a common tree in the PRD region indicates growth reduction in the late 1990s to mid-2000s coincident with increased air pollution, specifically at polluted sites compared to control sites [76].

An alternative, or parallel explanation for the reduced productivity in the 1995 to 2004 decade may be related to temperature stress, as He & Yang (2011) [77] observed that for the period 1996 to 2005, temperatures in PRD were above the 30-year average of 1981–2008 (and see also S1 Fig showing increased temperatures for Zhongshan and Zhuhai meteorological stations in PRD). This is supported by our findings in section 3.4 of negative correlations between growing season productivity indicators and temperature.

Studies in different parts of the world [58,59,78] and northern China [31] have reported an increase in growing season length due to an earlier SOS and /or delayed EOS, but our results did not indicate significant trends in SOS, EOS and LOS when the whole period 1982–2015 is considered. Most regions with increasing growing season length are non-tropical, where seasonal phenology is affected by freeze-thaw cycles, whereas nowhere in the PRD region is too cold for plant growth.

## Conclusion

In this study, we examined a 34-year trend in vegetation productivity, precipitation and temperature as well as the response of vegetation to seasonal climate indicators in the Pearl River Delta region of southern China. An overall increasing trend in productivity metrics was observed over the study period (1982–2015) which is more related to increasing temperature in autumn and winter, rather than to changes in the rainfall. This is contrary to the northern parts of China and other high latitude regions, where extension in growing season length due to advancement in green-up and/or delay in senescing dates is the primary factor for the higher seasonal productivity. In the PRD region, negative correlations between temperature and growing season productivity indicators for croplands, suggest that rising summer temperature during the critical crop development stage, have negative implications for rice production in the region. Some forested regions in the north of the PRD also show negative correlation with temperature for the main growing season productivity indicators. Although longer-term research is required to understand the impacts of warming on tropical forests, results suggest that increased warming would have adverse impacts on forests in the PRD region.

A reversal in the 3-decade trend of increasing growing season and peak productivity was observed between 1995 to 2004, accompanied by a period of decreased sunshine hours over PRD. This corresponds to a period of accelerated urbanisation and industrialisation, with aerosols contributing to solar dimming across the region, potentially with plant tissue damage from high pollutant concentrations. Along with this reduced radiation, higher summer temperatures during this period may explain the reverse productivity trend, as crops and forest species meet upper temperature thresholds.

The main finding of this study is overall increased vegetation productivity during the last 3 decades over the PRD associated with winter warming. However, our observed broad-scale, decade-long decline in productivity and negative correlations of summer growth with temperature, also have implications for crop production, the health of natural ecosystems and global climate modelling. The study presents the trends, but more research is needed to thoroughly explain these trends affecting this important region.

## Supporting information

S1 Fig Annual average time series of temperature observations recorded at the Zhongshan and Zhuhai meteorological station in the PRD. (DOCX)

S2 Fig Temporal trend in the monthly NDVI times series based on annual trend analysis. (DOCX)

S3 Fig Temporal trend in the annual phenometrics. Seasonal Adjusted (SA) removes first the seasonal cycle from a time series and then computes the trend on the seasonal-adjusted time series, Annual Aggregated Time Series (AAT) refers to trend based on annually aggregated time series, and the phenometrics trend is based on AAT as there one observation for each year.
(DOCX)

S4 Fig Land use and land cover-based graphical summary of the phenometrics. F = Forest, SH = Shrubland, CL = Cropland, Mix = Mosaic of Forest and Cropland.
(DOCX)

S5 Fig Trend in phenometrics. The metrics showing overall insignificant trends in most land cover classes of different land cover classes with loess fitting and confidence intervals; correlation coefficient (r), slope, significance (p) and total change (TC) is obtained by linear regression analysis of the variables over time.
(DOCX)

S6 Fig Maps of partial correlations of phenometrics and precipitation variables. (DOCX)

S7 Fig Maps of partial correlations of phenometrics and temperature variables. (DOCX)

S1 Table List of phenology dynamics metrics. (DOCX)
