# Supplementary material for: Trends in vegetation productivity related to climate change in China’s Pearl River Delta
Source: PLoS One. 2021 Feb 24;16(2):e0245467. doi: 10.1371/journal.pone.0245467 (PMC7904177; doi:10.1371/journal.pone.0245467)
Supplement: S1 Fig — (DOCX) [file pone.0245467.s001.docx]

**S1 Fig. Annual average time series of temperature observations recorded at the Zhongshan and Zhuhai meteorological station in the PRD.**
