# Supplementary material for: Trends in vegetation productivity related to climate change in China’s Pearl River Delta
Source: PLoS One. 2021 Feb 24;16(2):e0245467. doi: 10.1371/journal.pone.0245467 (PMC7904177; doi:10.1371/journal.pone.0245467)
Supplement: S3 Fig — Seasonal Adjusted (SA) removes first the seasonal cycle from a time series and then computes the trend on the seasonal-adjusted time series, Annual Aggregated Time Series (AAT) refers to trend based on annually aggregated time series, and the phenometrics trend is based on AAT as there one observation for each year. (DOCX) [file pone.0245467.s003.docx]

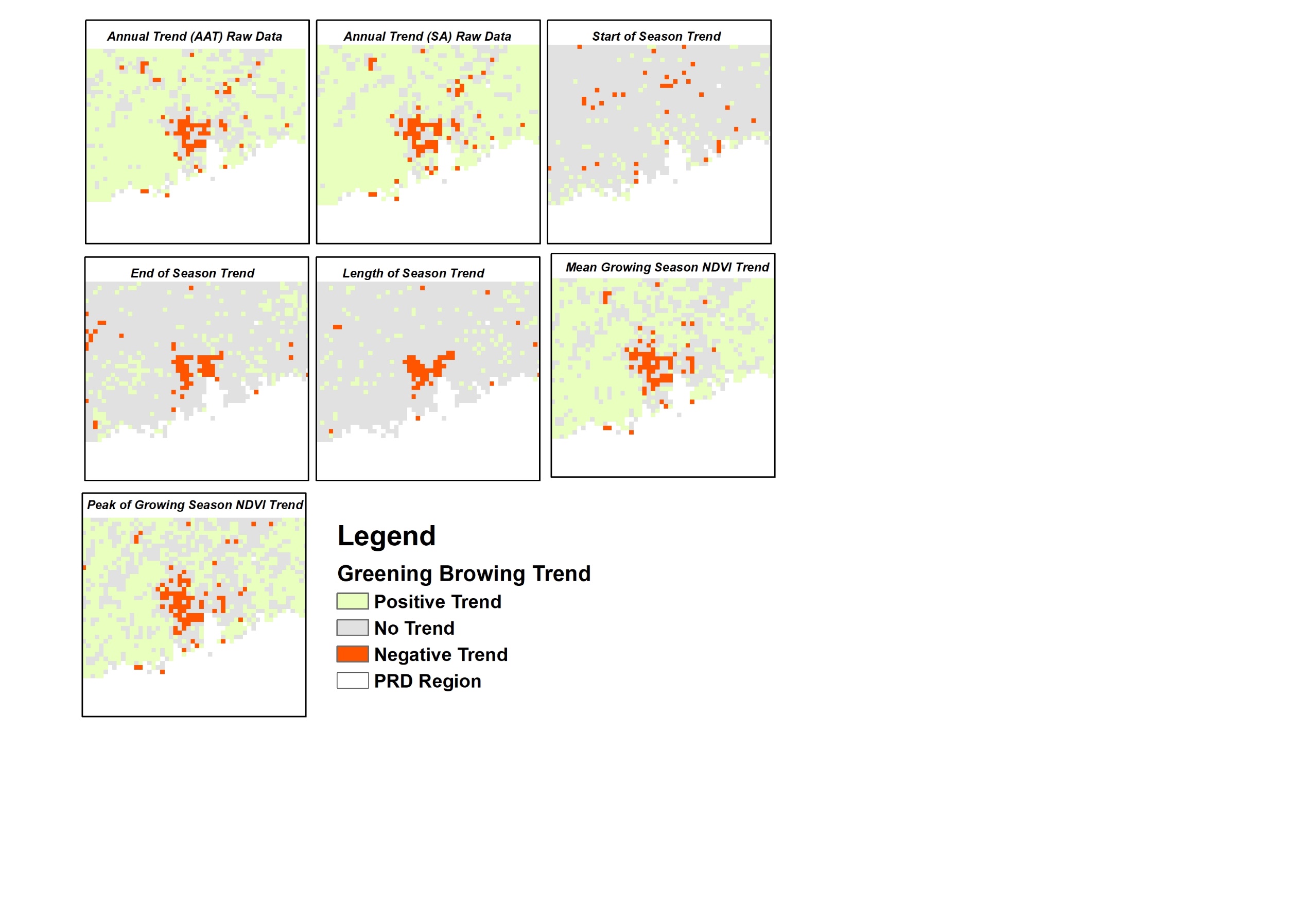


**S3 Fig. Temporal trend in the annual phenometrics**

Seasonal Adjusted (SA) removes first the seasonal cycle from a time series and then computes the trend on the seasonal-adjusted time series, Annual Aggregated Time Series (AAT) refers to trend based on annually aggregated time series, and the phenometrics trend is based on AAT as there one observation for each year.
