# Supplementary material for: Trends in vegetation productivity related to climate change in China’s Pearl River Delta
Source: PLoS One. 2021 Feb 24;16(2):e0245467. doi: 10.1371/journal.pone.0245467 (PMC7904177; doi:10.1371/journal.pone.0245467)
Supplement: S4 Fig — F = Forest, SH = Shrubland, CL = Cropland, Mix = Mosaic of Forest and Cropland. (DOCX) [file pone.0245467.s004.docx]

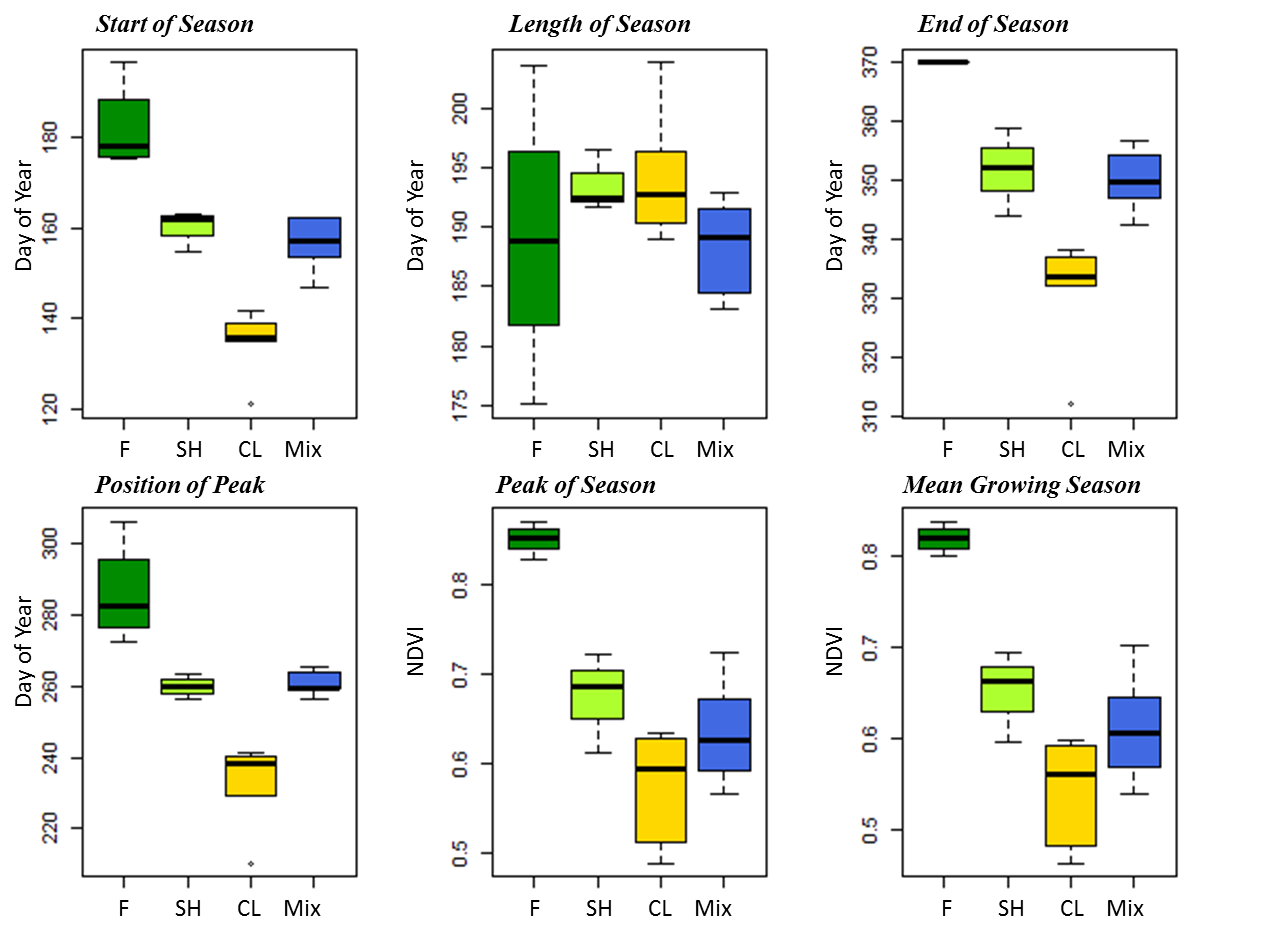


**S4 Fig. Land Use and Land Cover-based graphical summary of the phenometrics.**

F = Forest, SH = Shrubland, CL = Cropland, Mix = Mosaic of Forest and Cropland
