# Supplementary material for: Trends in vegetation productivity related to climate change in China’s Pearl River Delta
Source: PLoS One. 2021 Feb 24;16(2):e0245467. doi: 10.1371/journal.pone.0245467 (PMC7904177; doi:10.1371/journal.pone.0245467)
Supplement: S5 Fig — The metrics showing overall insignificant trends in most land cover classes of different land cover classes with loess fitting and confidence intervals; correlation coefficient (r), slope, significance (p) and total change (TC) is obtained by linear regression analysis of the variables over time. (DOCX) [file pone.0245467.s005.docx]

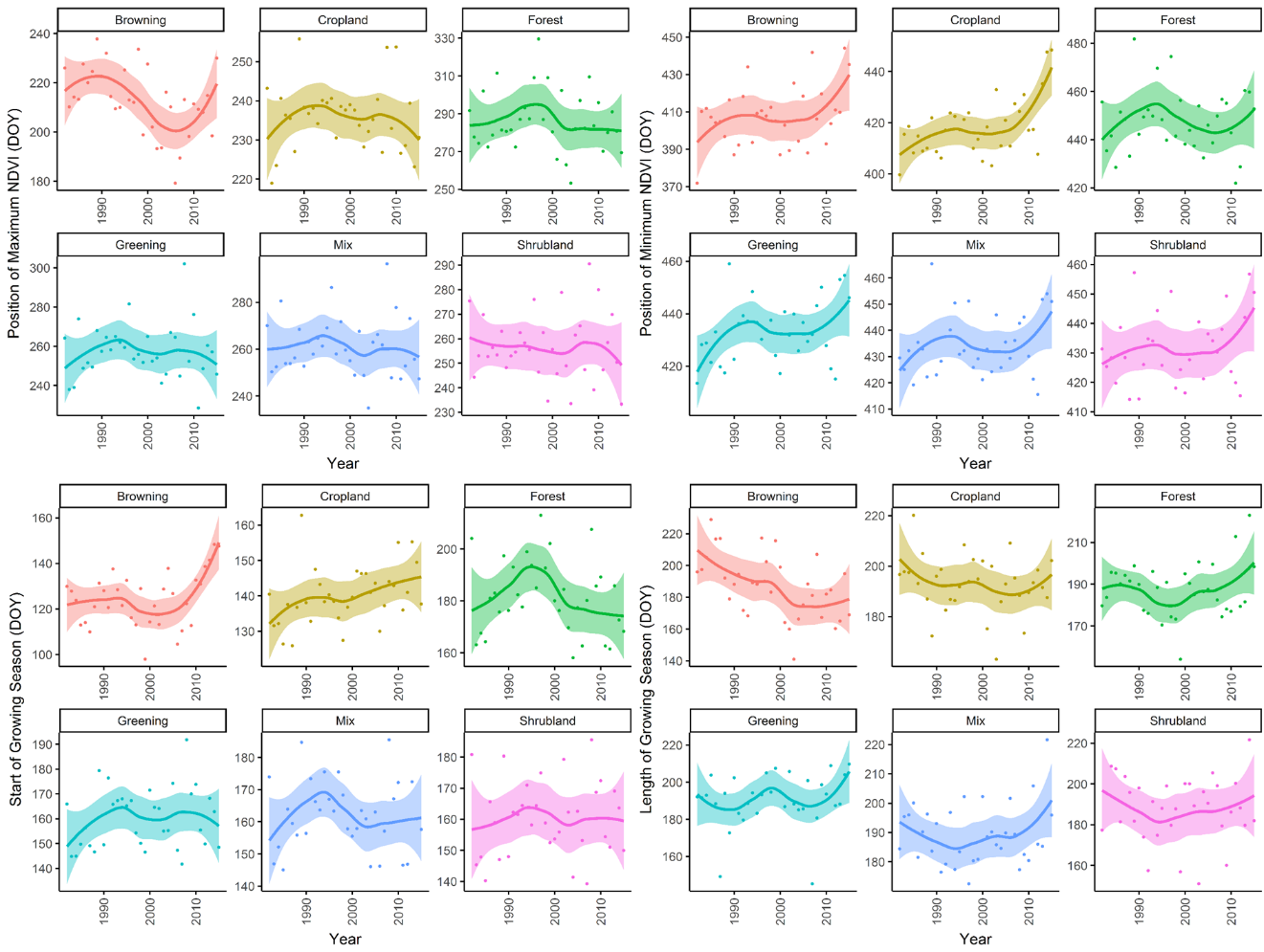


**S5 Fig. Trend in phenometrics**

The metrics showing overall insignificant trends in most land cover classes of different land cover classes with loess fitting and confidence intervals; correlation coefficient (r), slope, significance (p) and total change (TC) is obtained by linear regression analysis of the variables over time.
