# Supplementary figures and images for: Trends in vegetation productivity related to climate change in China’s Pearl River Delta
Source: PLoS One. 2021 Feb 24;16(2):e0245467. doi: 10.1371/journal.pone.0245467 (PMC7904177; doi:10.1371/journal.pone.0245467)

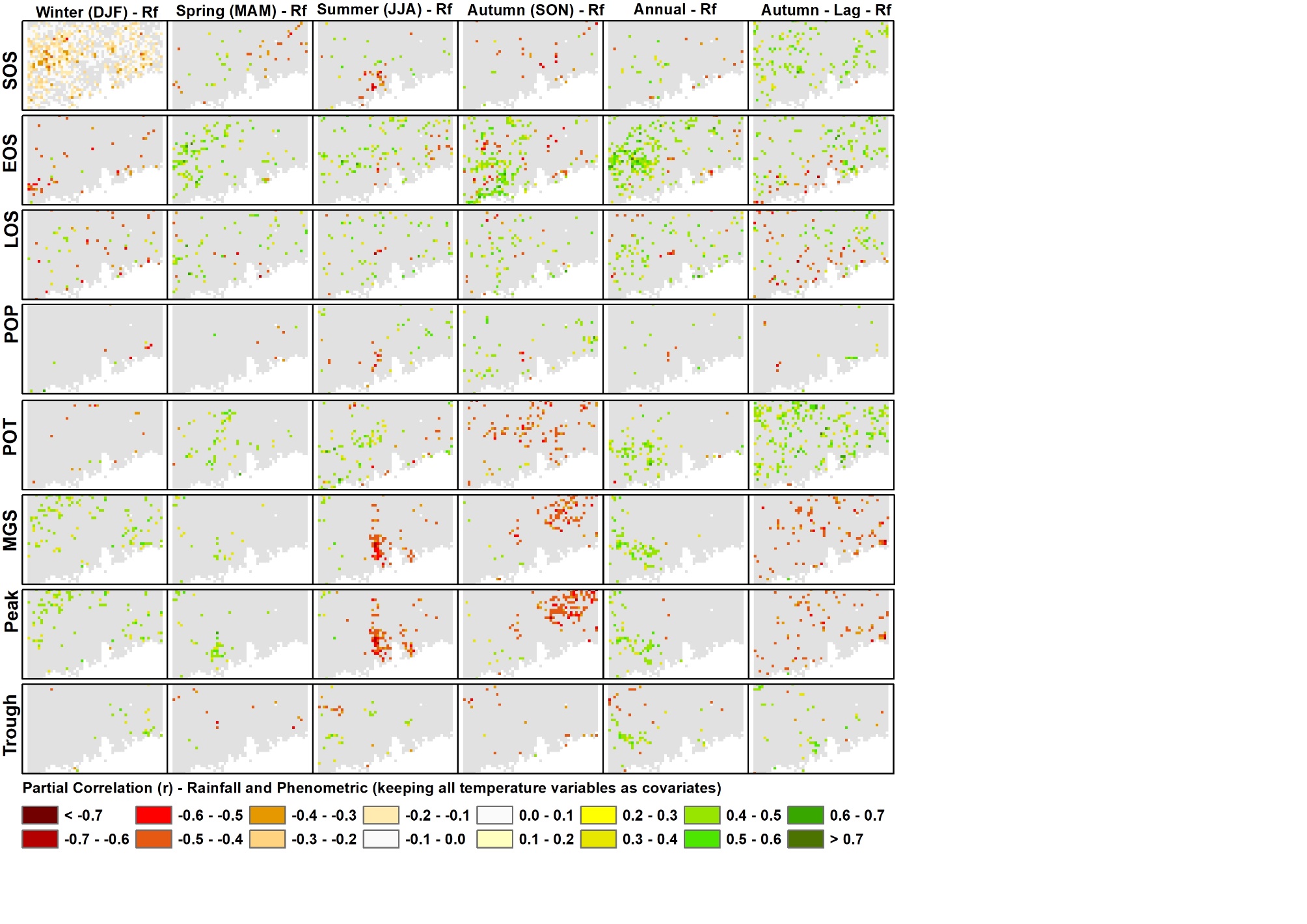


**S6 Fig. Maps of partial correlations of phenometrics and precipitation variables**

Supplement: S6 Fig — (DOCX) [file pone.0245467.s006.docx]

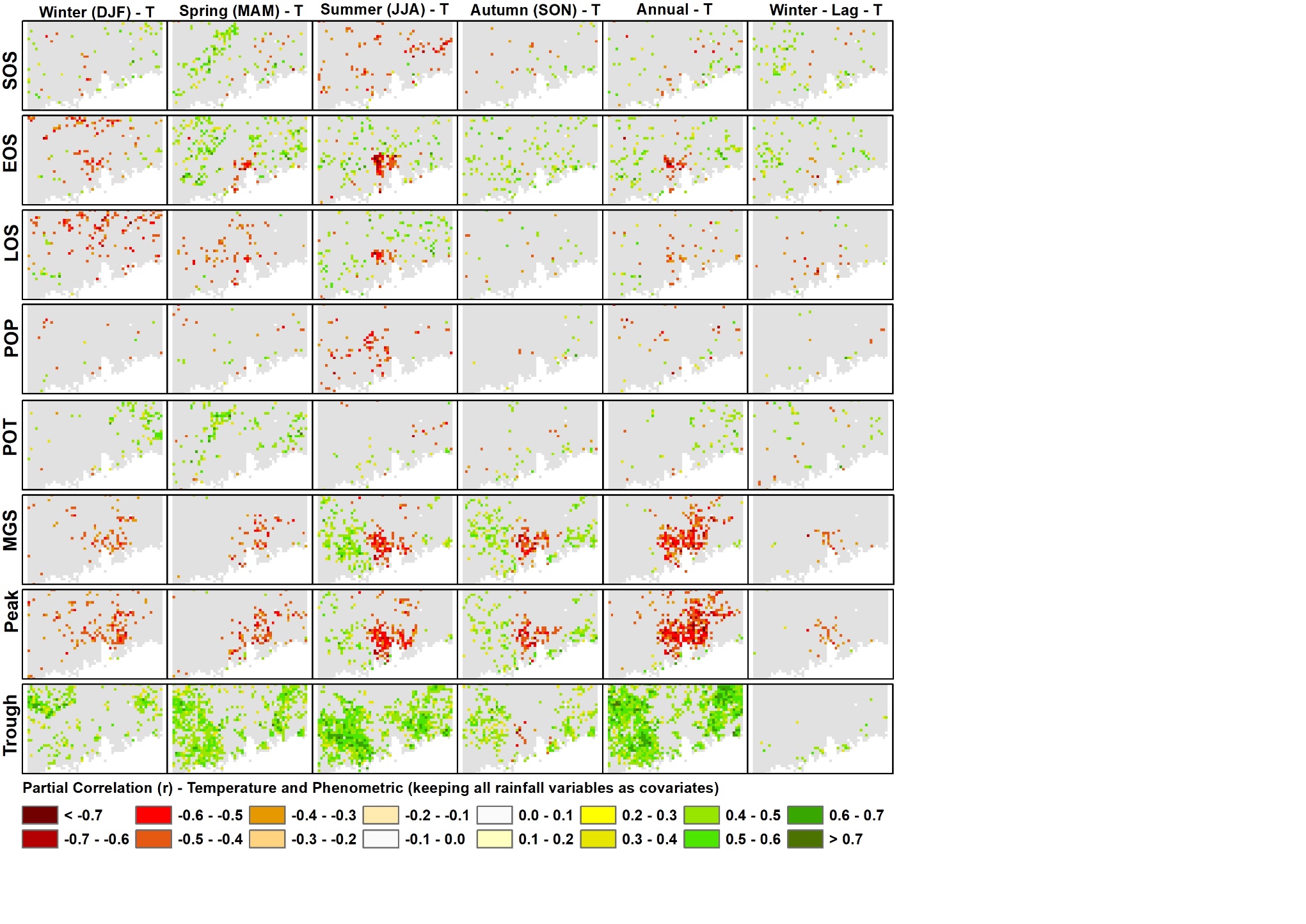


**S7 Fig. Maps of partial correlations of phenometrics and temperature variables**

Supplement: S7 Fig — (DOCX) [file pone.0245467.s007.docx]
