# Supplementary material for: Trends in vegetation productivity related to climate change in China’s Pearl River Delta
Source: PLoS One. 2021 Feb 24;16(2):e0245467. doi: 10.1371/journal.pone.0245467 (PMC7904177; doi:10.1371/journal.pone.0245467)
Supplement: S1 Table — (DOCX) [file pone.0245467.s008.docx]

**S1 Table. List of phenology dynamics metrics**

| **Phenology Metrics** | **Stands for** | **Description** | **Ecological Meaning** |
| --- | --- | --- | --- |
| SOS | Start of Growing Season | A time when NDVI ascends to the defined threshold value in spring | Approximates the start of the season: a time when green forage becomes available; time of highest quality forage |
| EOS | End of Growing Season | A time when NDVI descends to the defined threshold value in autumn | Approximates the end of the season: a time when seasonally active vegetation has effectively senesced or has been covered in snow; green forage becomes scarce (usually February to March) |
| LOS | Length of Growing Season | Number of days between green-up and senescence dates | Number of days when forage is available |
| Peak | Maximum Seasonal NDVI | Maximum NDVI value of the season | Proxy for maximum forage biomass of the season |
| Trough | Minimum Value of NDVI | Minimum Value of NDVI | Proxy for minimum forage biomass of the season |
| MGS | Mean Growing Season NDVI | Mean of all the NDVI values between SOS and EOS | Proxy of GPP |
| POP | Position of Peak | Time of maximum seasonal NDVI | Estimates of the time of maximum vegetation productivity in a growing season |
| POT | Position of Trough | Time of minimum seasonal NDVI in winter | Estimates the time of minimum vegetation productivity in an annual cycle of NDVI |
| GSNDVI | Mean Greening Season NDVI | Mean NDVI over a period of seasonal greening | Estimates the mean NDVI from June to October |
| BSNDVI | Mean Browning Season NDVI |  |  |
